# Supplementary material for: Room temperature electrofreezing of water yields a missing dense ice phase in the phase diagram
Source: Nat Commun. 2019 Apr 26;10:1925. doi: 10.1038/s41467-019-09950-z (PMC6486617; doi:10.1038/s41467-019-09950-z)
Supplement: Supplementary file 1 — Supplementary Information [file 41467_2019_9950_MOESM1_ESM.pdf]

Supplementary Information

Room temperature electrofreezing of water yields a missing dense ice phase in the phase diagram

Weiduo Zhu et al.

Supplementary Table 1. Fractional coordinates of the ice χ with orthogonal lattice (space group *Fdd*2; lattice constant: a = 24.3361 Å, b = 12.5284 Å, c = 4.3181 Å) based on vdW-DF2 calculation.

Atom	*X*	*Y*	*Z*	Occupancy
O	0.75	0.25	0.76185	1.0
O	0.78814	0.49681	0.01748	1.0
O	0.83208	0.15261	0.11117	1.0
O	0.91449	0.09511	0.68106	1.0
H	0.76131	0.52061	0.17382	1.0
H	0.81029	0.44361	0.13135	1.0
H	0.72543	0.29216	0.89838	1.0
H	0.84057	0.21762	0.23288	1.0
H	0.82067	0.09789	0.26312	1.0
H	0.88498	0.11507	0.82782	1.0
H	0.94617	0.07308	0.81019	1.0

Supplementary Table 2. Fractional coordinates of the polar ice B with orthogonal lattice (space group $P6_2$; lattice constant: a = 4.37119 Å, b = 5.05715 Å, c = 7.57112 Å) based on vdW-DF2 calculation.

Atom	*X*	*Y*	*Z*	Occupancy
O	0.25000	0.14730	0.25000	1.0
O	0.75000	0.14730	0.75000	1.0
O	0.75000	0.81397	0.25000	1.0
O	0.25000	0.81397	0.75000	1.0
O	0.50000	0.48064	0.00000	1.0
O	0.00000	0.48064	0.50000	1.0
H	0.18832	0.26586	0.34817	1.0
H	0.68832	0.26586	0.84817	1.0
H	0.92809	0.93253	0.23176	1.0
H	0.42809	0.93253	0.73176	1.0
H	0.38359	0.59920	-0.07992	1.0
H	-0.11641	0.59920	0.42008	1.0
H	0.31168	0.26586	0.15183	1.0
H	0.81168	0.26586	0.65183	1.0
H	0.57191	0.93253	0.26824	1.0
H	0.07191	0.93253	0.76824	1.0
H	0.61641	0.59920	0.07992	1.0
H	0.11641	0.59920	0.57992	1.0

Supplementary Methods

***Ab initio* MD Simulations.** To study the phase stability and possible water dissociation behaviour under intense electric field, the *ab initio* molecular dynamics (AIMD) simulation, as implemented in the Quantum-Espresso package[1], was adopted in the present work, following the previous study by Saittta *et al*.[2] but with a more accurate density functional.[3] The supercell used for AIMD contains 64 water molecules with the cubic lattice parameter of 12.59 Å, whereas the structural model of ice χ is its unit cell. The mesh of 1 × 1 × 1 and 1 × 1 × 3 k-points were used to sample the Brillouin zone of the bulk water and ice χ, respectively. The time step was set as 1 fs, and the simulation was carried out using the canonical ensemble (*NVT*) with the temperature being controlled at 270 K for bulk water and 100 K for ice χ. Two different external electric fields along *z* axis, 5 V nm^{-1} and 10 V nm^{-1}, were used to examine the phase stability and water dissociation behaviour. The GBRV pseudopotential of the element O and H with plane-wave cutoff of 40 Ry and a charge-density cutoff of 200 Ry were adopted for all the AIMD simulations.[3] The vdW-DF2 functional was employed to describe the van der Waals forces of water molecules.[4] The AIMD simulations run for about 160 - 10000 fs.

At 5.0 V nm^{-1}, no water dissociation was observed during 10000 fs simulation for the bulk water system at 270 K (see Figure 1(a) and Movie 1). In contrast, clear proton transfer phenomena can be observed at the stronger field of 10.0 V nm^{-1} (see Figure 1(b), 1(c) and Movie 2). A previous AIMD study based on the PBE functional showed that water molecules can be instantaneously dissociated under the intense field of 3.5-10 V nm^{-1} for bulk water[2]. Our results are consistent with the previous AIMD study.

Next, we examine whether water molecules in ice χ could dissociate under the intense electric field of 5.0 V nm^{-1} or 10.0 V nm^{-1} at 100 K. Within 10000 fs, dissociation of water molecules was not observed (see Figure 1(d) and Movie 3), whereas dissociation of water can be clearly seen within 1000 fs simulation time at the stronger field of 10.0 V nm^{-1} (see Figure 1(e) and Movie 4). Our AIMD simulations indicate that water molecule is unlikely to dissociate under electric field < 5.0 V nm^{-1}.

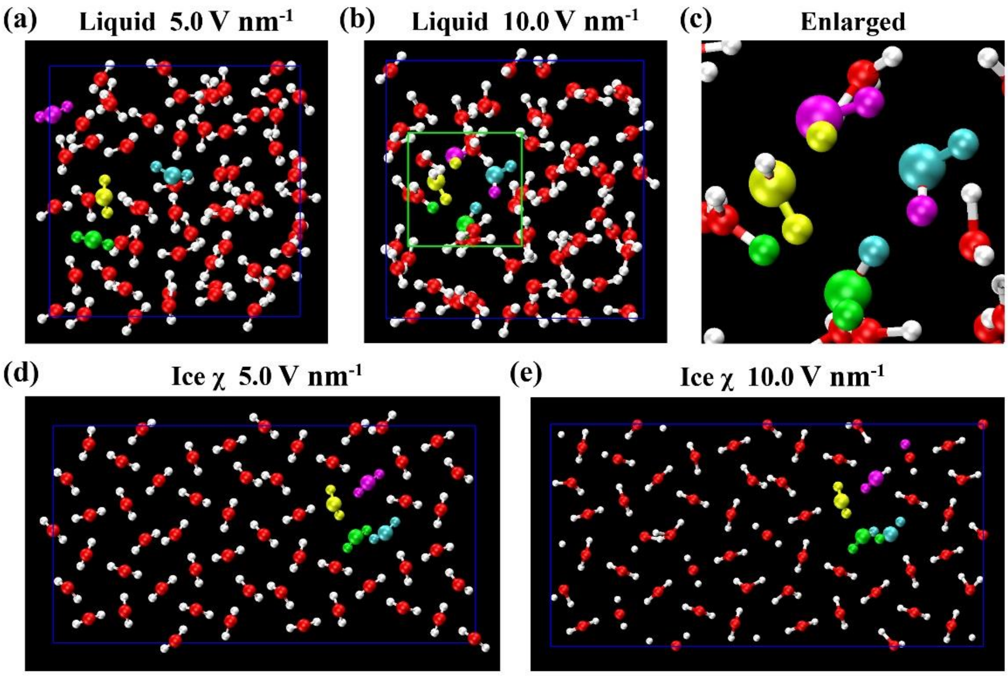

Supplementary Figure 1. (a) A snapshot of liquid water at T = 270 K and E = 5.0 V nm^{-1} after 10000 fs AIMD simulation. (b) A snapshot of typical proton transfer phenomena occurred in liquid water at T =270 K and E = 10.0 V nm^{-1}. (c) The enlarged image of the green box in (b). (d) A snapshot of ice χ at T = 100 K and E = 5.0 V nm^{-1} after 10000 fs AIMD simulation. (e) A snapshot of typical proton transfer phenomena for ice χ at T =100 K and E = 10.0 V nm^{-1}. The red balls represent oxygen atoms and the white balls represent hydrogen atoms. Four water molecules are marked with non-red/white color balls to highlight possible water dissociation event in the trajectory. Proton transfer events can be observed in both the liquid water and ice χ at E = 10.0 V nm^{-1}.

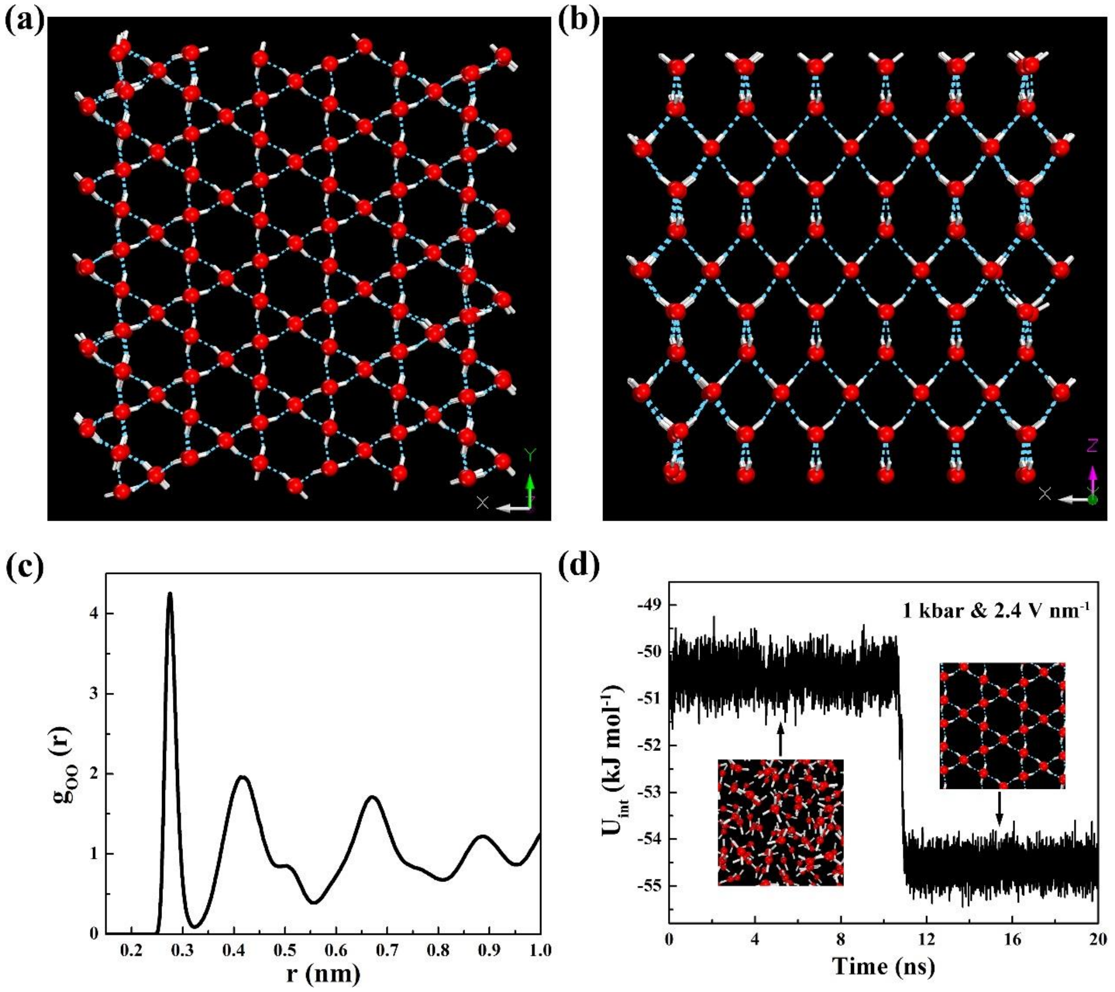

Supplementary Figure 2. Structure of polar ice B in (a) *z*-axis and (b) *y*-axis direction. (c) Computed radial distribution function (RDF) of oxygen atoms for the polar ice B. (d) Time-dependent potential energy per water molecule for the system at P = 1 kbar, T = 270 K and E = 2.4 V nm^{-1}. The red balls represent oxygen atoms, the white sticks represent hydrogen atoms, and blue dotted lines hydrogen bonds.

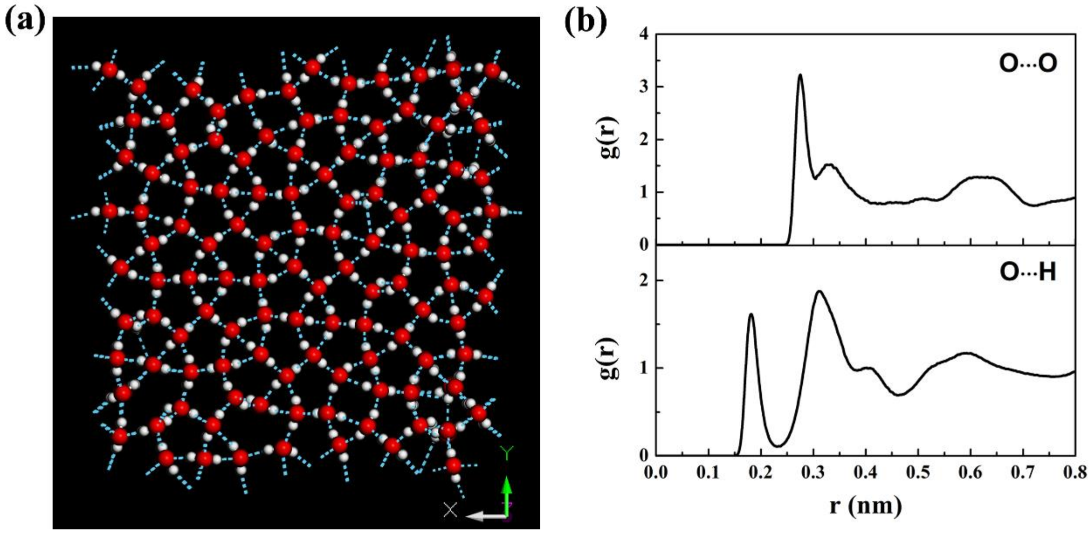

Supplementary Figure 3. Structure of the high-density ice in (a) *z*-axis direction. (b) O-O (oxygen-oxggen) and O-H (oxygen-hydrogen) RDFs of the high-density ice (10 kbar) at 270 K under electric field of 3.0 V nm^{-1}. The first neighbouring oxygen-hydrogen (O-H) coordination number for the amorphous ice is 2.02 ± 0.05, obtained by integrating the O-H RDF (exclude the O-H covalent bonds of the water molecule itself) data between 1.4 and 2.3 Å. This number reflects tetrahedral coordination and a fully hydrogen-bonded network for the amorphous ice[5].

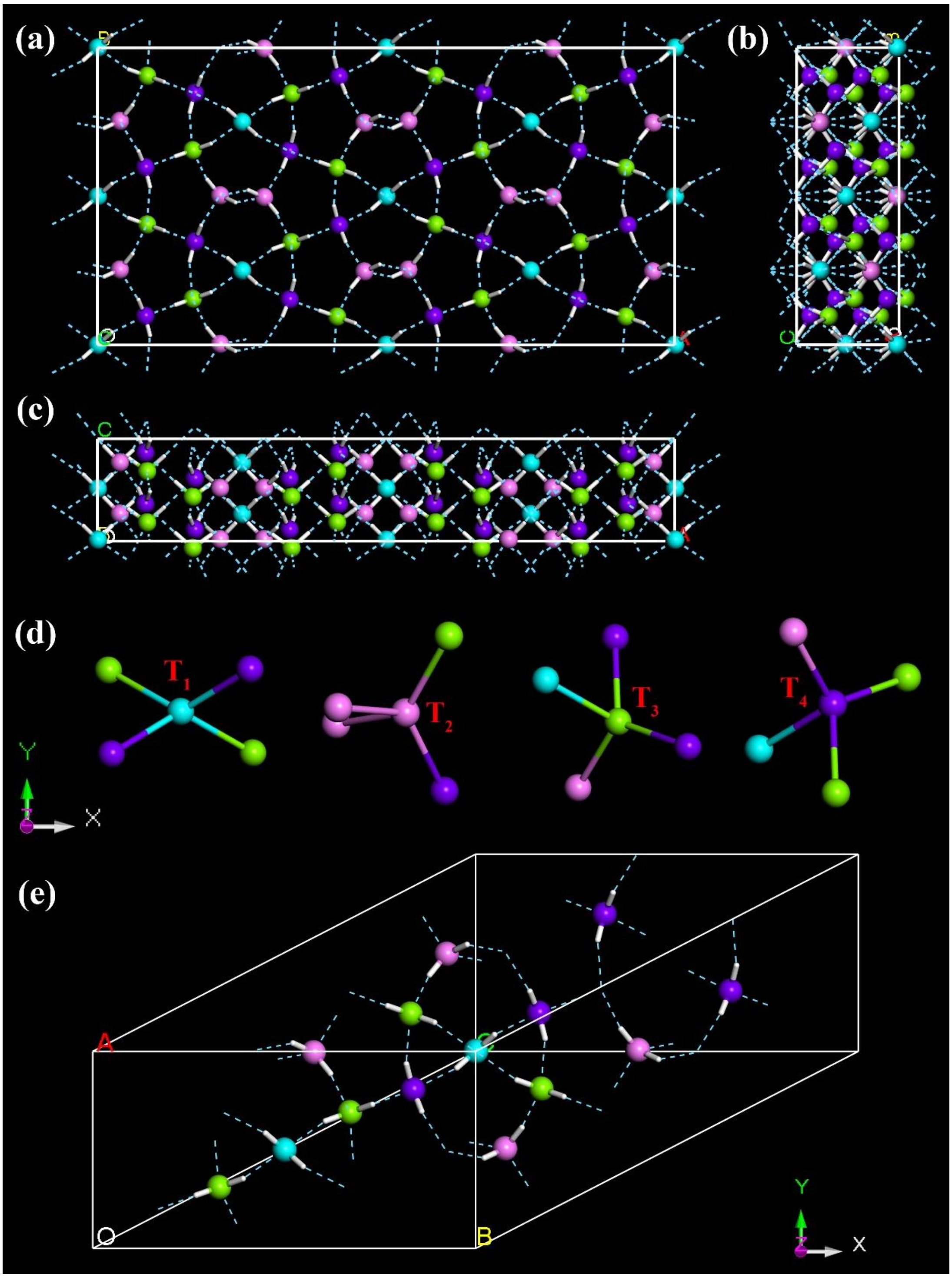

Supplementary Figure 4. The optimized unit cell of ice χ at the level of vdW-DF2, viewed in (a) *z*-axis direction, (b) *x*-axis direction, and (c) *y*-axis direction. (d) Four different local arrangements of water molecules, marked in cyan, pink, green, and purple, whose population ratio is 1: 2: 2: 2, respectively. (e) The primitive cell of ice χ. The red balls represent oxygen atoms, white sticks represent hydrogen atoms, and blue dotted lines represent hydrogen bonds.

We computed infrared spectrum of the ice χ for future experimental verification. As shown in Figure 5b, the high-frequency peak corresponding to the asymmetric stretching mode of O-H bond is at ~3311 cm^{-1}, while the peak corresponding to the symmetric stretching mode is at ~3213 cm^{-1}, while the corresponding peaks are at 3450 cm^{-1} and 3215 cm^{-1} in measured IR spectrum of liquid water[6]. The broad low-frequency peaks due to librational mode and H-O-H bending mode are at 802 cm^{-1} and 1601 cm^{-1}, respectively. The measured H-O-H bending mode for liquid water at 298 K is at ~1650 cm^{-1} as shown from a previous experiment[7].

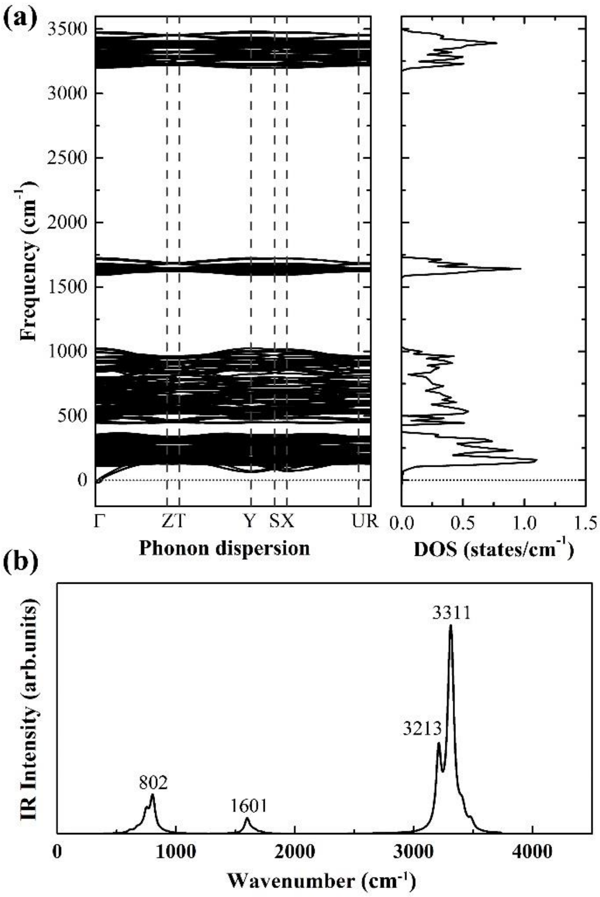

Supplementary Figure 5. (a) Computed phonon dispersion of ice χ using the DFPT[8] method implemented in VASP 5.4. Definition of special points in the Brillouin zone: Γ-(0,0,0), Z-(0,0,0.5), T-(-0.5,0,0.5), Y-(-0.5,0,0), S-(-0.5,0.5,0), X-(0,0.5,0), U-(0,0.5,0.5), R-(-0.5,0.5,0.5). Note that the low imaginary frequencies correspond to the acoustic frequencies at Gamma point likely due to the translational invariance being violated in the DFPT calculations, originated from the discreteness of the FFT grid. (b) Infrared spectrum of the ice χ from DFPT calculation.

To examine relative stability of ferroelectric ice χ to neighbouring ice phases in the phase diagram, we also calculate their enthalpies under different pressures at 0 K by using the strongly constrained and appropriately normed (SCAN)[9] density functional. The calculations are done with VASP 5.4 code with hardest projector-augmented wave (PAW) pseudopotentials and the 1000 eV plane-wave basis set cut off[10]. The number of *k* points are chosen so that the spacing in the *k* point grid in each direction of reciprocal space is within 0.02 $Å^{-1}$ to 0.04 $Å^{-1}$ for all the ice phases considered.

The results are consistent with vdW-DF2[3] computation, demonstrating that ice χ is one of the most stable very-high-density ices in the high-pressure region at zero temperature, between ice II and ice VI. As Figure 6 shows, a low-density ice XI is the most stable phase in the region of 0 kbar < P < 3.78 kbar. The higher-density ice II becomes more favorable between 3.78 kbar and 12.05 kbar. For P > 12.05 kbar, the ferroelectric ice χ becomes the most stable polymorph. For $P \geqslant$ 21.53 kbar, the ice VI becomes more stable than ice χ.

In addition, we compute the zero-point energy (ZPE) correction at the harmonic level (based on vdw-DF2 functional) and then add the ZPE correction to the free energy. As shown in Figure 7, the calculation results indicate that ZPE correction does not change the predicted relative stability among the ices considered, but only slightly change the stability region. The ice χ still appears in the high-pressure region as the most stable phase between ice II and ice VI. Hence, we expect that the quantum nuclear motion would have little effect on the predicted relative stability among the ices considered.

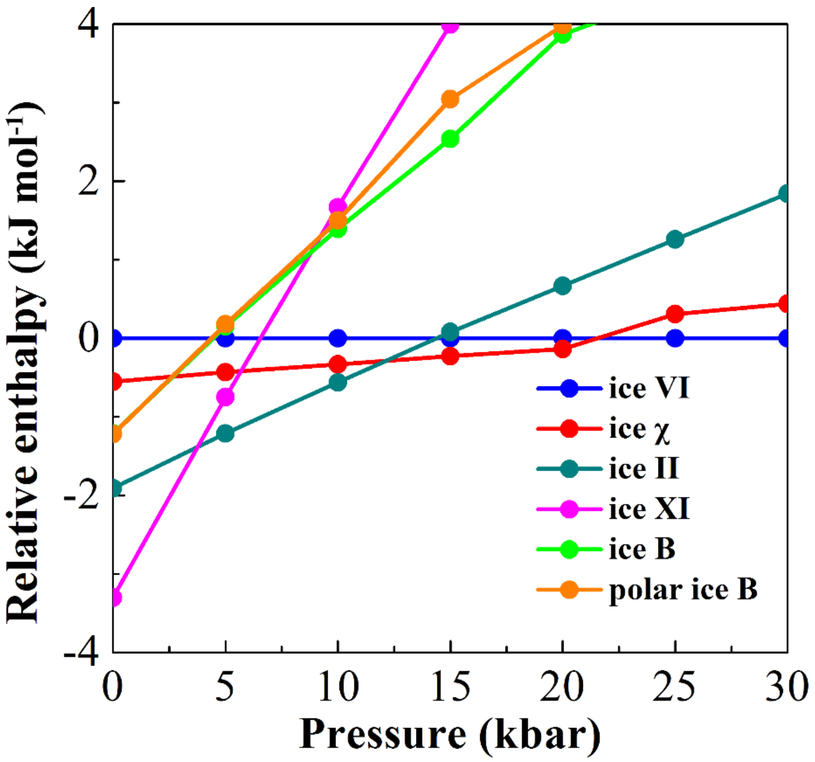

Supplementary Figure 6. Relative enthalpy per water molecule (based on SCAN functional calculations) versus pressure for the ice χ, ice II, ice XI, ice B, and polar ice B, respectively, where the ice VI is taken as the reference.

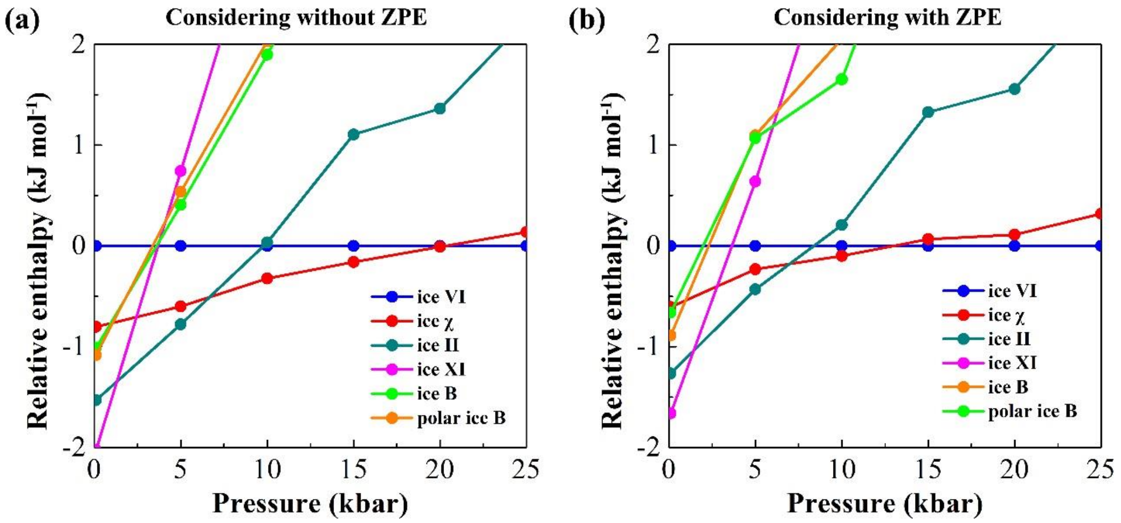

Supplementary Figure 7. Relative enthalpy per water molecule (calculated based on vdw-DF2 functional) *versus P* for ice χ, ice II, ice XI, ice B, and polar ice B, where ice VI is taken as the reference: (a) Without ZPE, and (b) with ZPE correction.

Here, given the possibility of the disorder of hydrogen atoms for ice χ, we present nine randomly generated ice structures based on the same oxygen atom occupancies of ice χ but with different orientations of hydrogen atoms (see Figure 8 below). To examine relative stability of the ferroelectric ice χ with respect to the nine hydrogen-disordered ice structures, we calculate their enthalpies under different pressures at 0 K based on vdw-DF2 calculations. As Figure 9 shows, ice χ is still the most stable structure among them, although their enthalpy differences are very small (less than 0.4 kJ mol^{-1}).

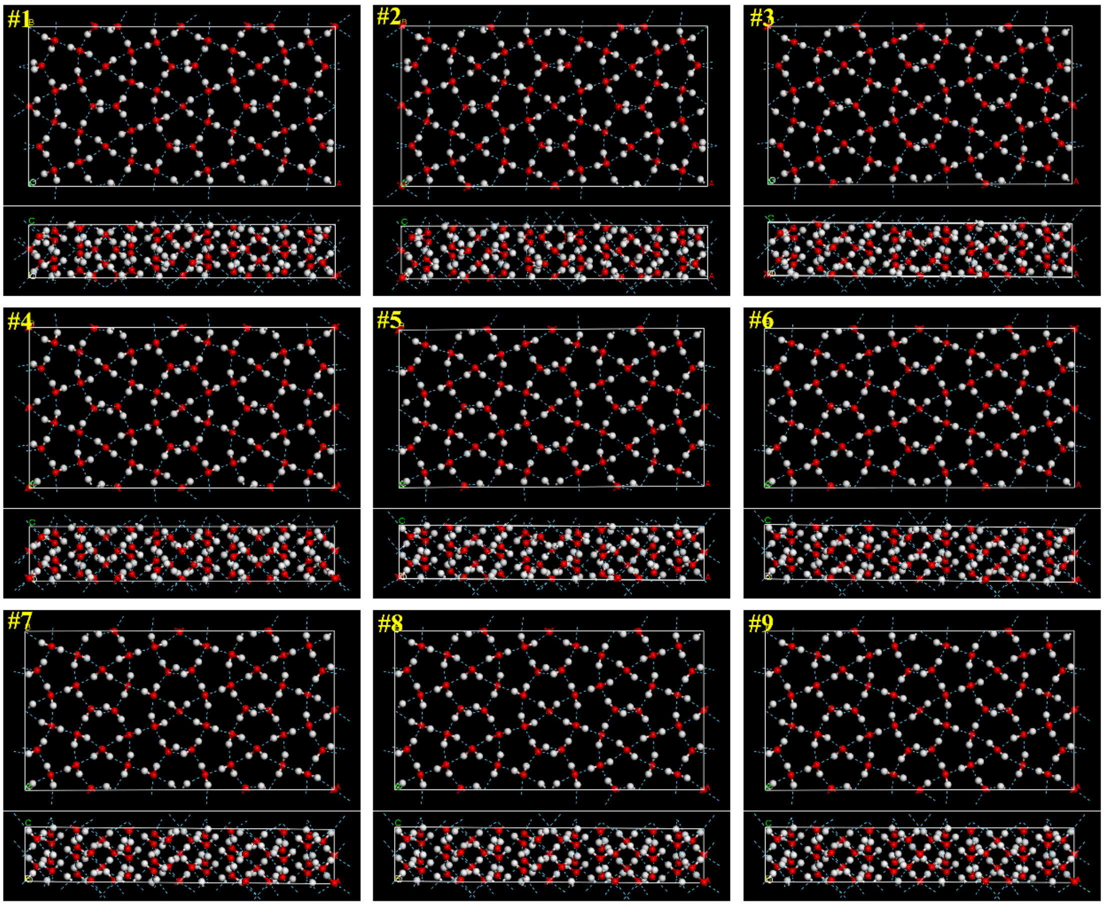

Supplementary Figure 8. Top view and side view of nine randomly generated ice χ structures with the same oxygen atom occupancies as ice χ and different hydrogen atom orientations. Oxygen atoms are depicted as red balls, hydrogen atoms as white balls, and hydrogen bonds as blue dotted lines.

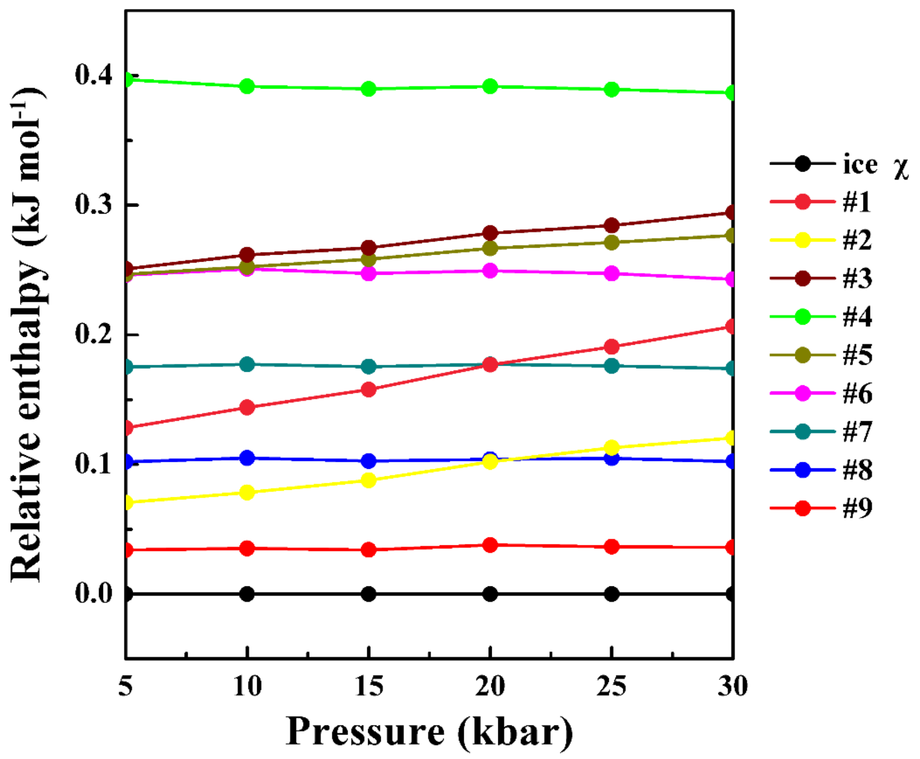

Supplementary Figure 9. Relative enthalpy per water molecule (based on vdw-DF2 calculations) versus pressure for ice χ and the nine randomly generated ice structures with different hydrogen orientations, where ice χ is taken as the reference.

Supplementary References

1. Giannozzi, P.; Baroni, S.; Bonini, N.; Calandra, M.; Car, R.; Cavazzoni, C.; Ceresoli, D.; Chiarotti, G. L.; Cococcioni, M.; Dabo, I., QUANTUM ESPRESSO: a modular and open-source software project for quantum simulations of materials, *J. Phys. Condens. Matter* **21**, 395502 (2009).
2. Saitta, A. M.; Saija, F.; Giaquinta, P. V., Ab initio molecular dynamics study of dissociation of water under an electric field, *Phys. Rev. Lett.* **108**, 207801 (2012).
3. Garrity, K. F.; Bennett, J. W.; Rabe, K. M.; Vanderbilt, D., Pseudopotentials for high-throughput DFT calculations, *Comp. Mater. Sci.* **81**, 446-452 (2014).
4. Lee, K.; Murray, E. D.; Kong, L. Z.; Lundqvist, B. I.; Langreth, D. C., Higher-accuracy van der Waals density functional, *Phys. Rev. B* **82**, 081101 (2010).
5. Finney, J. L.; Bowron, D. T.; Soper, A. K.; Loerting, T.; Mayer, E.; Hallbrucker, A., Structure of a new dense amorphous ice, *Phys. Rev. Lett.* **89**, 205503 (2002).
6. Gopalakrishnan, S.; Liu, D.; Allen, H. C.; Kuo, M.; Shultz, M. J., Vibrational spectroscopic studies of aqueous interfaces: salts, acids, bases, and nanodrops, *Chemical reviews* **106**, 1155-75 (2006).
7. Brubach, J. B.; Mermet, A.; Filabozzi, A.; Gerschel, A.; Roy, P., Signatures of the hydrogen bonding in the infrared bands of water, *J. Chem. Phys.* **122**, 184509 (2005).
8. Refson, K.; Tulip, P. R.; Clark, S. J., Variational density-functional perturbation theory for dielectrics and lattice dynamics, *Phys. Rev. B* **73**, 155114 (2006).
9. Sun, J.; Remsing, R. C.; Zhang, Y.; Sun, Z.; Ruzsinszky, A.; Peng, H.; Yang, Z.; Paul, A.; Waghmare, U.; Wu, X.; Klein, M. L.; Perdew, J. P., Accurate first-principles structures and energies of diversely bonded systems from an efficient density functional, *Nat. Chem.* **8**, 831-6 (2016).
10. Santra, B.; Klimes, J.; Tkatchenko, A.; Alfe, D.; Slater, B.; Michaelides, A.; Car, R.; Scheffler, M., On the accuracy of van der Waals inclusive density-functional theory exchange-correlation functionals for ice at ambient and high pressures, *J. Chem. Phys.* **139**, 154702 (2013).
