# Supplementary material for: Room temperature electrofreezing of water yields a missing dense ice phase in the phase diagram
Source: Nat Commun. 2019 Apr 26;10:1925. doi: 10.1038/s41467-019-09950-z (PMC6486617; doi:10.1038/s41467-019-09950-z)
Supplement: Supplementary file 3 — Description of Additional Supplementary Files [file 41467_2019_9950_MOESM3_ESM.pdf]

Description of Additional Supplementary Files

File Name: Supplementary Movie 1
Description: An AIMD simulation trajectory of bulk water at 270 K and 5.0 V nm-1 (bulk_5v.avi).

File Name: Supplementary Movie 2
Description: An AIMD simulation trajectory of bulk water at 270 K and 10.0 V nm-1 (bulk_10v.avi).

File Name: Supplementary Movie 3
Description: An AIMD simulation trajectory of ice χ at 100 K and 5.0 V nm-1 (ice_5v.avi).

File Name: Supplementary Movie 4
Description: An AIMD simulation trajectory of ice χ at 100 K and 10.0 V nm-1 (ice_10v.avi).

File Name: Supplementary Movie 5
Description: A classical MD simulation trajectory of the liquid to solid phase transition for ice χ (chi.avi).

File Name: Supplementary Movie 6
Description: A classical MD simulation trajectory of the liquid to solid phase transition for polar ice B (polarB.avi).
